# Study on Extraction Valuable Metal Elements by Co-Roasting Coal Gangue with Coal Gasification Coarse Slag

**DOI:** 10.3390/molecules29010130

**Published:** 2023-12-25

**Authors:** Jincheng Zhao, Tao Yu, Huan Zhang, Yu Zhang, Lanting Ma, Jinling Li, Chengtun Qu, Te Wang

**Affiliations:** 1 Yan’an Key Laboratory of Low Carbon Synergistic Control Technology and Reservoir Protection for Oil and Gas Field Environmental Pollution, Shaanxi Fuquan Environmental Protection Technology Co., Ltd., Yan’an 727500, China; 21211070886@stumail.xsyu.edu.cn (J.Z.); zhuan@wnu.edu.cn (H.Z.); zhangyu@xynu.edu.cn (Y.Z.); malanting2020@xsyu.edu.cn (L.M.); xianquct@xsyu.edu.cn (C.Q.); 2 State Key Laboratory of Petroleum Pollution Control, College of Chemistry and Chemical Engineering, Xi’an Shiyou University, Xi’an 710065, China; lijinling@xsyu.edu.cn (J.L.); 21212070982@stumail.xsyu.edu.cn (T.W.); 3 Shaanxi Oil and Gas Pollution Control and Reservoir Protection Key Laboratory, College of Chemistry and Chemical Engineering, Xi’an Shiyou University, Xi’an 710065, China

**Keywords:** co-roasting, H_2_SO_4_ leaching, phase transformation, Al, Fe extraction

## Abstract

Coal gangue (CG) and coal gasification coarse slag (CGCS) possess both hazardous and resourceful attributes. The present study employed co-roasting followed by H_2_SO_4_ leaching to extract Al and Fe from CG and CGCS. The activation behavior and phase transformation mechanism during the co-roasting process were investigated through TG, XRD, FTIR, and XPS characterization analysis as well as Gibbs free energy calculation. The results demonstrate that the leaching rate of total iron (TFe) reached 79.93%, and Al^3+^ achieved 43.78% under the optimized experimental conditions (co-roasting process: CG/CGCS mass ratio of 8/2, 600 °C, 1 h; H_2_SO_4_ leaching process: 30 wt% H_2_SO_4_, 90 °C, 5 h, liquid to solid ratio of 5:1 mL/g). Co-roasting induced the conversion of inert kaolinite to active metakaolinite, subsequently leading to the formation of sillimanite (Al_2_SiO_5_) and hercynite (FeAl_2_O_4_). The iron phases underwent a selective transformation in the following sequence: hematite (Fe_2_O_3_) → magnetite (Fe_3_O_4_) → wustite (FeO) → ferrosilite (FeSiO_3_), hercynite (FeAl_2_O_4_), and fayalite (Fe_2_SiO_4_). Furthermore, we found that acid solution and leached residue both have broad application prospects. This study highlights the significant potential of co-roasting CG and CGCS for high-value utilization.

## 1. Introduction

Coal gangue (CG) is a solid waste generated in the process of coal mining and washing, constituting approximately 10–15% of the total coal production [1,2]. Currently, China’s coal gangue reserves amount to around 7 billion tons, with its production continuously increasing each year, making it one of the largest industrial wastes [3,4]. Coal gasification slag (CGS) is a solid waste generated by the coal gasification process. It primarily consists of residual materials derived from raw coal, which undergo a series of processes including crushing, melting, sintering, gasification, deposition, and accumulation. Ultimately, CGS comprises residues that have not undergone redox and gasification reactions. Depending on the particle size, it can be further classified into two categories: coal gasification coarse slag (CGCS) and coal gasification fine slag (CGFS) [5]. The coal gasification process consumes approximately 100 million tons of coal annually, with the national production of CGS exceeding 60 million tons per year and showing exponential growth [6]. The deposition of CG and CGS typically occurs in open piles on vacant land. Despite the distance from urban areas, the accumulation of substantial quantities of coal-based waste residues still engrosses significant land resources and poses a grave ecological threat. The environmental concerns encompass air pollution caused by dust, water pollution, and soil contamination from heavy metals (Cu, Cd, Cr, Pb, Zn, Hg, and Sn), as well as various geological hazards [7,8]. Additionally, the oxidative spontaneous combustion during stacking of CG and CGS results in the emission of detrimental gases such as CO_2_, CO, H_2_S, SO_2_, and NO_x_ [9]. In addition, both CG and CGS exhibit high concentrations of silicon, aluminum, iron, and titanium. The primary constituents of CG include quartz, clay minerals, and carbonaceous materials [10]. On the other hand, the mineral composition of CGS is predominantly composed of amorphous alumino-silicates, crystalline phase minerals, and residual carbon. Amorphous alumino-silicates account for over 60% of the total composition while quartz and calcite dominate the crystalline phase minerals [11].

At present, the comprehensive utilization of CG and CGS is primarily concentrated in areas such as combustion and power generation, construction materials, fertilizers, chemical product extraction and preparation, boiler blending, and ecological restoration [12,13,14]. However, the comprehensive utilization rate of both CG and CGS in China significantly lags behind that of developed countries. Therefore, it is strategically imperative to undertake extensive utilization of CG and CGS. Leaching is a widely used method for metal extraction and can be performed using various types of solvents and reagents. It involves dissolving the metal from the raw material into a liquid solution, which can then be further processed to isolate the metal. Leaching represents an energy-efficient process that can be optimized to minimize waste generation and mitigate environmental impact. Nevertheless, it is crucial to consider the potential environmental consequences associated with any extraction method and opt for the most environmentally friendly alternative whenever feasible.

Currently, the main methods regarding the extraction of aluminum from coal gangue (CG) and coal gasification slag (CGS) are alkali and acid methods [15,16]. Alkali methods, including sintering and leaching [16], have been extensively studied for the extraction of alumina from CG and CGS. However, the sintering method requires high temperatures (around 1100 °C to 1200 °C), resulting in increased energy consumption and significant emission of dicalcium silicate residue [17]. In addition, the alkali leaching method enables the alumina in CG and CGS to transform into a sodium aluminate solution, while iron and titanium become insoluble residue, resulting in a waste of resources. Additionally, alkaline solutions dissolve silica in CG and CGS, necessitating an additional desilication process [18]. Consequently, the application of alkali methods for aluminum extraction is not promising.

Compared with alkali methods, acid extraction offers competitiveness and suitability for treating CG and CGS due to its advantages such as a shorter process duration, lower energy consumption, easy separation of silica and aluminum components, as well as thorough separation. Although there are fewer studies on iron extraction from CG and CGS at present time, common methods include acid leaching extraction or reductive roasting-magnetic separation. Reduction roasting-magnetic separation involves roasting under a reducing atmosphere (e.g., H_2_ [19,20], methane, or natural gas [21]), which facilitates rapid conversion of hematite to magnetite in CG and CGS followed by magnetic separation to obtain magnetite. Similar to acid extraction of aluminum, acid extraction of iron also possesses these advantages. However, it should be noted that acid leaching extraction usually requires pre-activation when extracting iron and aluminum from CG and CGS.

Various methods, including mechanical activation and thermal activation (roasting activation, and microwave activation) [22,23,24], have been reported as effective pre-activation approaches for acid leaching to extract the available components in CG and CGS. However, a single activation method often fails to achieve efficient extraction, leading to the adoption of composite activation methods in current research for extracting its active ingredients [25,26]. Qin et al. [23] employed a combination of mechanical grinding, low-temperature roasting, and deionized water leaching to extract aluminum and other trace elements from CG. The fully ground CG was sieved through a 100-mesh sieve and mixed according to the mass ratio of CG:NH_4_Cl:(NH_4_)_2_SO_4_ = 1:2:1. Subsequently, the mixture was subjected to roasting at 400 °C for 1 h in a muffle furnace. The roasted sample was placed in a conical flask of 20 mL of 0.4 m deionized water and shaken at 60 °C for 1 h. The final leaching yields of Al, Li and Fe were 56.35%, 80.83% and 32.77%, respectively. Han et al. [27] used an acid-base combination method, wherein a 2.0 mol/L KOH solution was utilized as an activator to immerse the activated CG in a hydrochloric acid solution (2.0 mol/L) for 2 h. The resulting acid leach solution was subsequently evaporated at 110 °C for 2 h, yielding extraction rates of Al^3+^ and Si^4+^ after drying that reached as high as 78.9% and 69.2%, respectively. Shao et al. [2] proposed a recovery technology using a combination of thermal activation + HNO_3_ leaching to treat CG. Following milling, the CG was subjected to thermal activation at 550 °C for 0.5 h in a muffle furnace. Under optimized leaching conditions (150 °C, theoretical HNO_3_ dosage, liquid-solid ratio of 5:1 mL/g, and 2 h), extraction rates of aluminum, gallium, lithium, and iron were achieved at 95.2%, 56.4%, 80.5%, and 2.1%, respectively. Additionally, flotation is also a valuable method to upgrade solid particles before leaching [28,29], which can not only improve the efficiency and quality of aluminum and iron extraction by acid leaching, but also minimizes the process cost and environmental pollution. Moreover, it finds extensive application in the efficient recovery of minerals, solid waste, as well as heavy metal ions present in water.

In these methods, the leaching rate of valuable metal elements is influenced not only by process conditions but also by the composition of CG and CGS. These existing techniques are difficult to directly replicate and apply. Furthermore, most studies focus on single coal-based waste residues such as CG, CGS, and fly ash, with limited research on extracting valuable metal elements from two or more coal-based waste residues. Therefore, it is necessary to explore a leaching process condition that can simultaneously extract valuable metal elements from both CG and CGS. It should be noted that both CG and CGCS contain fixed carbon which can act as a reducing agent during the reduction roasting process. Based on current research progress, an innovative approach involving co-roasting and H_2_SO_4_ leaching was proposed for extracting valuable metal elements (Al, Fe) from CG and CGCS. This study addresses the gap in synergistic extraction of valuable metal elements from both CG and CGCS; compared to separate extraction methods, it improves the leaching rate of valuable metal elements while reducing energy consumption and H_2_SO_4_ dosage. The aim of this method is to provide technical support for achieving synergistic extraction of valuable metal elements from two or more coal-based waste residues.

## 2. Results and Discussion

### 2.1. Optimization of Co-Roasting Parameter

The influence of CGCS content on the extraction efficiency of valuable metal elements was investigated by thoroughly mixing CG with CGCS in a specific ratio, followed by flat placement in a corundum crucible and co-roasting in a tubular pyrolysis furnace at 700 °C for 1 h. Simultaneously, the leaching conditions were set as follows: leaching temperature of 50 °C, H_2_SO_4_ concentration of 30 wt%, liquid to solid ratio of 5:1 mL/g, and leaching time of 5 h.

**Figure 1:**
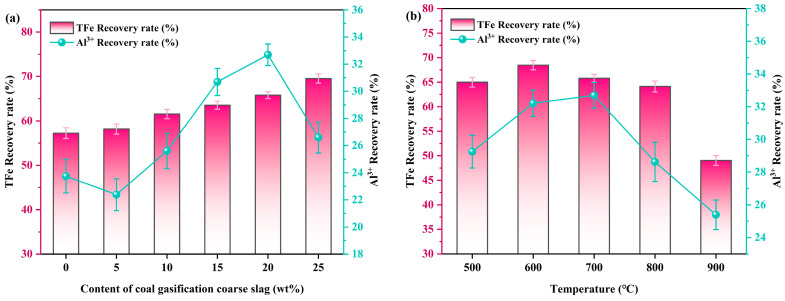
(**a**) The effect of different CGCS content on the leaching rates of Al^3+^ and TFe, (**b**) the effect of different co-roasting temperature on the leaching rates of Al^3+^ and TFe (co-roasting process: 1 h; H_2_SO_4_ leaching process: 30 wt% H_2_SO_4_, 50 °C, 5 h, liquid to solid ratio of 5:1 mL/g).

As depicted in Figure 1a, in the absence of CGCS addition, the leaching rates of Al^3+^ and TFe were merely 23.74% and 57.22%, respectively. With an increase in CGCS content, the leaching of Al^3+^ exhibited a non-linear trend with an initial decrease followed by an increase before declining again, reaching its peak at 32.69% for a content of 20 wt%. Conversely, the leaching rate of TFe gradually increased and reached its maximum value of 69.52% when the CGCS content was at 25 wt%. This behavior can be attributed to the lower percentage of Al^3+^ content compared to that of TFe within CGCS, resulting in an enhanced release of TFe as more CGCS is added. Additionally, the decrease in the extraction rate of Al^3+^ can be attributed to the relatively low initial Al content in the CGCS sample compared to CG. As the amount of CGCS added increases, there is a gradual reduction in the overall Al content in the mixed raw material, ultimately leading to a decline in the extraction rate of Al^3+^. Furthermore, there was a notable rise in carbon content which facilitated sufficient CO generation for hematite (Fe_2_O_3_) reduction. Considering both the high proportion of CG present in the co-roasting mixture and elevated aluminum concentration within CG itself, it was deemed appropriate to select a CGCS addition level at 20 wt%.

As shown in Figure 1b, the effect of co-roasting temperature on the extraction of valuable metal elements was investigated by incorporating 20 wt% CGCS, while keeping other conditions such as liquid to solid ratio constant. With the increase in co-roasting temperature, the leaching rates of Al^3+^ and TFe initially exhibited an upward trend followed by a decline. At 600 °C, the leaching rate of TFe reached a peak of 68.47%, indicating that the reduction effect of co-roasting was optimized at this time. This can be attributed to the temperature-induced promotion of hematite (Fe_2_O_3_) reduction to magnetite (Fe_3_O_4_) and wustite (FeO). However, as the temperature continued to rise, the leaching rate of TFe gradually decreased due to potential formation of other substances that hindered iron phase reduction in the co-roasting process. The leaching rates of Al^3+^ were measured at 32.21% and 32.69% at temperatures of 600 °C and 700 °C, respectively, which could be attributed to metakaolinite becoming inert material beyond 700 °C resulting in minimal or even negative impact on leaching rates’ enhancement. Therefore, considering both leaching rates’ performance and economic factors, a co-roasting temperature of 600 °C was selected.

### 2.2. Raw Material Characterization

**Figure 2:**
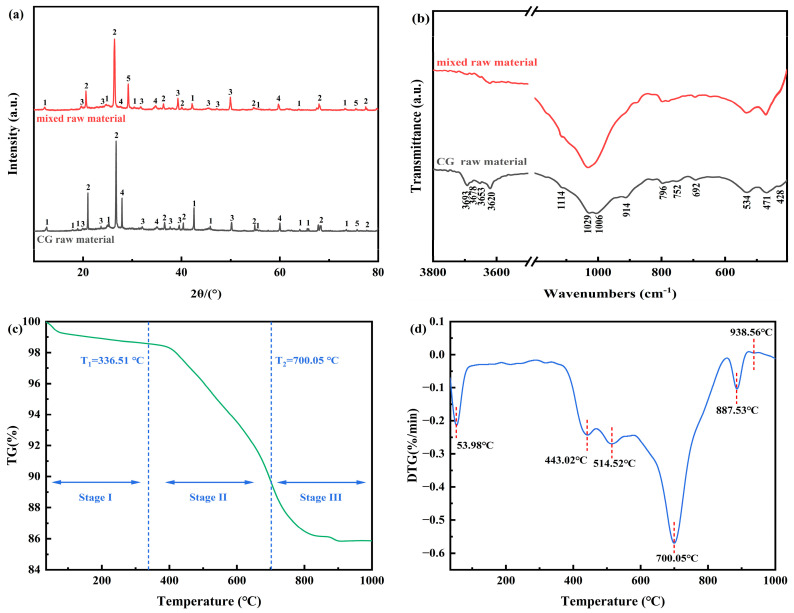
(**a**) XRD patterns of CG raw material, mixed raw material; 1—kaolinite; 2—quartz; 3—hematite; 4—muscovite; 5—calcite; (**b**) FTIR spectra of CG raw material and mixed raw material, (**c**,**d**) TG and DTG curves of co-roasting process.

The XRD pattern of CG raw material and mixed raw material are presented in Figure 2a, revealing predominant mineral constituents including kaolinite, quartz, hematite, muscovite, and calcite. Figure 2b displays the FTIR spectra of CG raw material and mixed raw material. The bands observed at 914, 3620 cm^−1^ and 3653, 3678, 3693 cm^−1^ can be attributed to the internal and external OH stretching vibrations in the layered structure of kaolinite octahedron [30,31]. Additionally, characteristic bands corresponding to Al-O-Si and Al-O vibrations in kaolinite were identified at 534 and 692 cm^−1^, respectively [32,33]. Furthermore, absorption peaks at 428 and 1114 cm^−1^ along with double peaks at 1029 and 1006 cm^−1^ correspond to the Si-O vibration in kaolinite. Lastly, absorption peaks observed at 796 and 752 cm^−1^ are indicative of the Si-O-Si stretching vibration in kaolinite [34,35].

The co-roasting of coal gangue and coal gasification coarse slag was divided into three stages based on the variations in TG and DTG curves, as shown in Figure 2c,d. Stage I occurred at an onset temperature of 35 °C–336.51 °C, accompanied by moisture evaporation. Stage II, ranging from 336.51 °C to 700.05 °C, involved the release of volatiles, carbon burning, and observed structural hydroxyl desorption of kaolinite [31,36]. This stage exhibited a maximum DTG peak with a value of 0.569%/min at a corresponding temperature of 700.05 °C. Stage III existed within the range of 700.05 °C–1000 °C and displayed two DTG peaks at temperatures of 887.53 °C and 938.56 °C, respectively; this finding may be attributed to macromolecular organic matter degradation, fixed carbon decomposition, and mullite formation [37].

### 2.3. Phase Transformation during Co-Roasting Process

**Figure 3:**
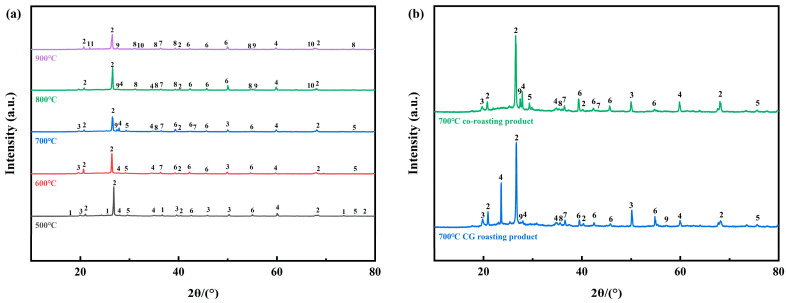
(**a**) XRD patterns of 500 °C–900 °C co-roasting products; (**b**) XRD patterns of 700 °C CG roasting product, 700 °C co-roasting products; 1—kaolinite; 2—quartz; 3—hematite; 4—muscovite; 5—calcite; 6—magnetite; 7—wustite; 8—fayalite; 9—sillimanite; 10—hercynite; 11—ferrosilite.

The XRD patterns of the co-roasting products obtained at different co-roasting temperatures are presented in Figure 3a. At 500 °C, the diffraction peaks of kaolinite still persist but with reduced intensity compared to the mixed raw material, indicating partial decomposition of kaolinite [38]. Upon increasing the temperature from 500 °C to 600 °C, the diffraction peaks of kaolinite disappear completely, suggesting successful transformation from inert kaolinite to active metakaolinite. Notably, active metakaolinite appears amorphous and does not exhibit any discernible features in XRD analysis [39]. Additionally, some hematite diffraction peaks vanish at 500 °C while a new magnetite phase is detected.

Further increasing temperature to 600 °C, a significant decomposition of hematite occurred, leading to an increase in the diffraction peaks of magnetite. Meanwhile, the presence of wustite was observed following co-roasting at 600 °C. Subsequently, at temperatures ranging from 700 °C–900 °C, distinct diffraction peaks corresponding to fayalite, sillimanite, hercynite, and ferrosilite were identified [40,41,42,43]. This observation elucidates why the reduction of iron phases during co-roasting was impeded and resulted in a minimal or even decreased leaching rate of Al^3+^. Notably, at a co-roasting temperature of 800 °C, calcite underwent decomposition while muscovite remained remarkably stable even when subjected to elevated temperatures up to 900 °C.

Figure 3b displays the XRD patterns of the CG roasting product and co-roasting product at 700 °C. As depicted in the figure, compared to the solely CG roasting product at 700 °C, the diffraction peaks of hematite are weaker while those of magnetite are stronger in the co-roasting product at 700 °C, and there is an increase in wustite’s number of diffraction peaks. This can be attributed to a higher carbon content in mixed raw material that provides a better reducing atmosphere and promotes further reduction of iron phases [44,45]. Additionally, there are more diffraction peaks for sillimanite in CG roasting products than co-roasting products which result in a lower leaching rate for Al^3+^.

The aforementioned results demonstrate that the temperature increase and carbon presence contribute to the reduction of iron phase in the co-roasting process. However, excessively high temperatures lead to sintering of wustite with Si and Al in the mixed raw material, resulting in the formation of other iron phases which impede iron phase reduction. Simultaneously, as temperature increases, metakaolinite reacts with Si and Fe in the mixed raw material to form sillimanite and hercynite, consequently reducing the leaching rate of Al^3+^.

**Figure 4:**
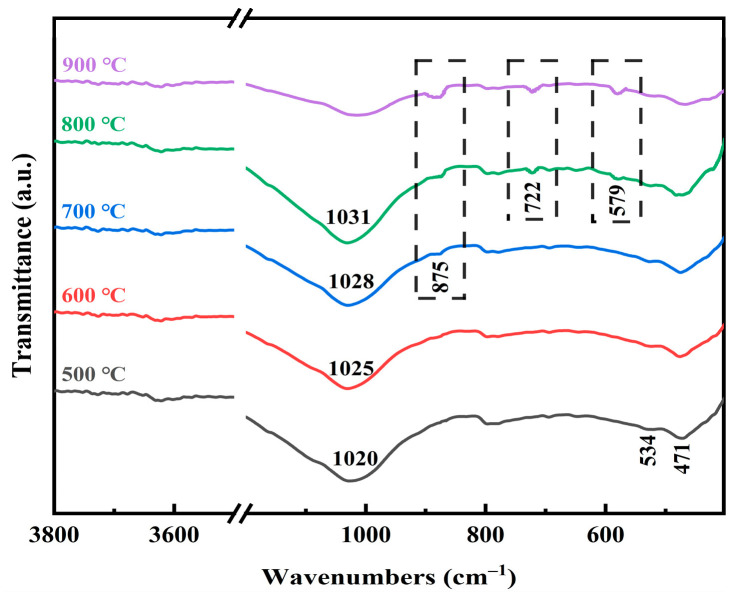
FTIR patterns of 500 °C–900 °C co-roasting product.

The co-roasting products were characterized using FTIR analysis. As depicted in Figure 4, at a co-roasting temperature of 500 °C, the gradual removal of the hydroxyl group from kaolinite resulted in the absence of absorption peaks associated with OH stretching vibrations (3693, 3678, 3653, 3620, and 914 cm^−1^), indicating the initiation of kaolinite transformation into metakaolinite [18,31]. Simultaneously, a decrease in the intensity of Al-O-Si stretching vibration at 534 cm^−1^ suggested that the dehydroxylation process was accompanied by the disruption of Al-O-Si bonds and exacerbation of crystal defects.

With the further increase in co-roasting temperature, the Si-O vibration peaks at 1006 and 1029 cm^−1^ merge into a broadband peak at 1020 cm^−1^, which subsequently shifts to shorter wavelengths between 500 °C–800 °C. This shift is attributed to the enhanced orderliness of the silicate structure [45]. During the transformation of kaolinite into metakaolinite, as metakaolinite gradually loses its ordering and average bond angle increases within the Si-O-Si network, the stretching vibration peak at 471 cm^−1^ assigned to Si-O-Si weakens and broadens with increasing temperatures from 500 °C–800 °C [46].

Significantly, the adsorption bond located at 875 cm^−1^ associated with inert sillimanite (Al_2_SiO_5_) was detected after co-roasting at temperatures ranging from 700 °C to 900 °C [45]. Furthermore, the characteristic peaks of hercynite (FeAl_2_O_4_) related to Fe-O at 579 cm^−1^ and Al-O bond at 722 cm^−1^ were observed after co-roasting within the temperature range of 800 °C–900 °C [47]. These findings are consistent with the XRD results, providing further evidence for the formation of new inert components contributing to the decrease in leaching rates of Al^3+^ and TFe.

**Figure 5:**
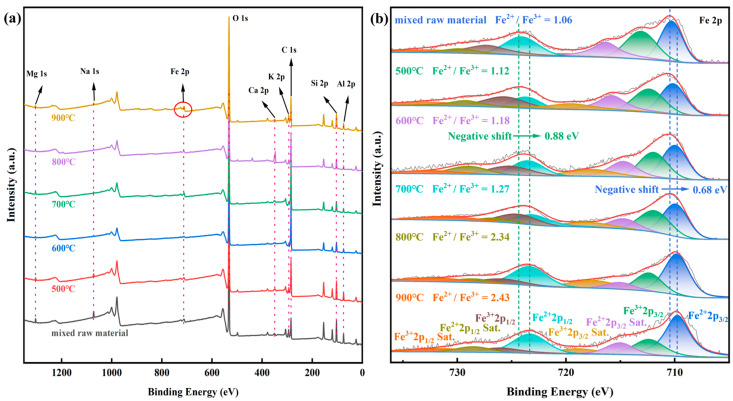
XPS analysis (**a**) survey scan XPS spectra for mixed raw material, and 500 °C–900 °C co-roasting products; XPS survey of Fe2p for (**b**) mixed raw material, and 500 °C–900 °C co-roasting products.

In addition to XRD and FTIR, XPS analysis was conducted to characterize the mixed raw material and co-roasting products. The effect of co-roasting on the atomic valence characteristics of mineral surfaces was investigated through XPS analysis in Figure 5. Figure 5a illustrates the comprehensive survey scans of the six samples, revealing that Si, Al, Fe, O, C, K, Ca, Mg, and Na were the predominant elements at the surface. These findings align with the chemical composition and proximate analysis of raw materials.

In Figure 5b, the high-resolution XPS survey of Fe2p for these six samples reveals that the Fe2p3/2 peak exhibits a narrower width, higher intensity, and larger area compared to the Fe2p1/2 peak due to spin-orbit (j-j) coupling [48,49,50]. The Fe2p spectrum of the mixed raw material indicates that the two photoelectron peaks at binding energies of 726.9 eV (Fe2p1/2) and 712.79 eV (Fe2p3/2) can be attributed to Fe^3+^, which appears to correspond with the presence of Fe^3+^ in the hematite phase [51,52]. Additionally, the observed photoelectron peaks related to Fe(II) minerals in trace amounts seem to represent contributions from Fe^2+^ species.

According to Figure 5b, the Fe^2+^ content of the co-roasting product at 500 °C exhibited an increase, with the ratio of Fe^2+^/Fe^3+^ rising from 1.06 to 1.12. Additionally, lower binding energy revealed a low intensity peak of Fe^2+^, indicating the transformation of hematite after co-roasting and contributing to the presence of magnetite in the product at 500 °C [51,53]. This suggests that reduction from the Fe^3+^ state to Fe^2+^ has occurred [54]. Moreover, these findings align with those obtained from XRD analysis as evidenced by satellite peaks (714.98 eV and 729.18 eV) observed in the co-roasting product at 600 °C for Fe^2+^. The ratio of Fe^2+^/Fe^3+^ further increased to 1.18 during this stage. Since magnetite does not exhibit satellite peaks [49,55], it can be inferred that these satellite peaks are attributed to wustite based on XRD analysis results, signifying that the transition from magnetite to wustite has already taken place. Specifically, with the increase in co-roasting temperature, the binding energy positions of the maxima of the Fe2p3/2 and Fe2p1/2 peaks were shifted from 710.4 eV and 724.2 eV to 709.72 eV and 723.32 eV, with negative shifts of 0.68 eV and 0.88 eV, respectively, compared to the mixed raw material. Moreover, there was an increase in the Fe^2+^/Fe^3+^ ratio to 2.43, indicating a change in the chemical environment where more divalent iron is present (i.e., alterations in types and quantities of elements bound to it [56]). Combining these findings with XRD analyses, it can be attributed that within the co-roasting product at temperatures ranging from 700 °C–900 °C, Fe^2+^ originates from two sources: a minor contribution from magnetite formation and transformation of wustite into fayalite, hercynite, and ferrosilite phases.

The XPS results mentioned above provide robust evidence supporting the transformation of hematite phase into magnetite, wustite, fayalite, hercynite, and ferrosilite phases during the co-roasting process. Additionally, this phase transformation process resulted in a reduction in both TFe and Al^3+^ leaching rates.

### 2.4. Co-Roasting Reaction Mechanism

**Figure 6:**
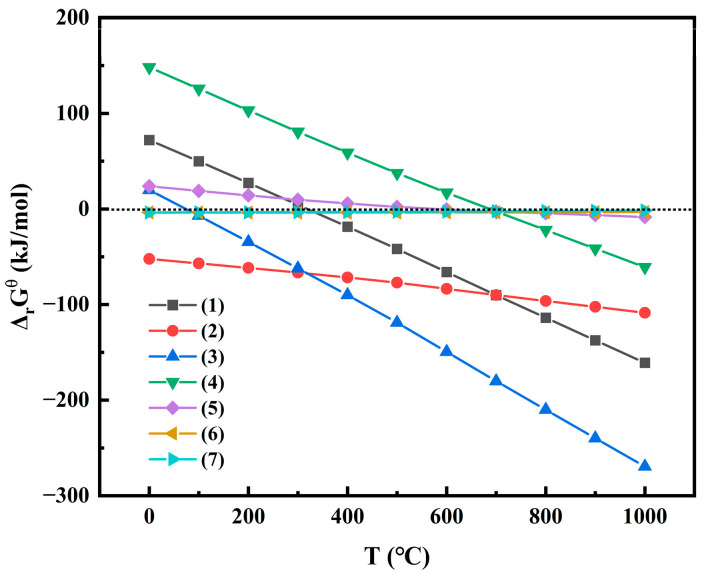
Diagram of Gibbs free energy (Δ_r_G^θ^) versus temperature for reactions occurring during the co-roasting process of mixed raw material.

The reactions that may occur during the co-roasting process of mixed raw material are enumerated as Equations (1)–(11) [57,58]. Thermodynamic calculations for some of the reactions were performed using HSC 9.0 software, and the temperature dependency of Δ_r_G^θ^ is illustrated in Figure 6.

(1) $$3{\mathrm{Fe}}_{2}O_{3}\;(s)+C\;(s)\;\text{→}\;2{\mathrm{Fe}}_{3}O_{4}\;(s)+\mathrm{CO}\;(g)$$

(2) $$3{\mathrm{Fe}}_{2}O_{3}\;(s)+\mathrm{CO}\;(g)\;\text{→}\;2{\mathrm{Fe}}_{3}O_{4}\;(s)+CO_{2}\;(g)$$

(3) $$6{\mathrm{Fe}}_{2}O_{3}\;(s)+C\;(s)\;\text{→}\;4{\mathrm{Fe}}_{3}O_{4}\;(s)+CO_{2}\;(g)$$

(4) $${\mathrm{Fe}}_{3}O_{4}\;(s)+C\;(s)\;\text{→}\;3\mathrm{FeO}\;(s)+\mathrm{CO}\;(g)$$

(5) $${\mathrm{Fe}}_{3}O_{4}\;(s)+\mathrm{CO}\;(g)\;\text{→}\;3\mathrm{FeO}\;(s)+CO_{2}\;(g)$$

(6) $$\mathrm{FeO}\;(s)+{\mathrm{Al}}_{2}O_{3}\;(s)\;\text{→}{\mathrm{FeAl}}_{2}O_{4}\left(s\right)$$

(7) $$\mathrm{FeO}\;(s)+\mathrm{Si}O_{2}\;(s)\;\text{→}\;\mathrm{FeSi}O_{3}\left(s\right)$$

(8) $$2\mathrm{FeO}\;(s)+\mathrm{Si}O_{2}\;(s)\;\text{→}{\mathrm{Fe}}_{2}\mathrm{Si}O_{4}\left(s\right)$$

(9) $${\mathrm{Al}}_{2}O_{3}\cdot 2\mathrm{Si}O_{2}\cdot 2H_{2}O\;(s)\;\text{→}{\mathrm{Al}}_{2}O_{3}\cdot 2\mathrm{Si}O_{2}\;(s)+2H_{2}O\;(g)$$

(10) $${\mathrm{Al}}_{2}O_{3}\cdot 2\mathrm{Si}O_{2}\left(s\right)+0.5{\mathrm{Fe}}_{2}O_{3}\left(s\right)+0.5C\;\left(s\right)\text{→}{\mathrm{FeAl}}_{2}O_{4}\left(s\right)+2\mathrm{Si}O_{2}\left(s\right)+0.5\mathrm{CO}\;(g)$$

(11) $$2[{\mathrm{Al}}_{2}O_{3}\cdot 2\mathrm{Si}O_{2}]\;(s)\;\text{→}\;0.5{\mathrm{Al}}_{2}\mathrm{Si}O_{5}\left(s\right)+1.5\mathrm{Si}O_{2}\;(s)+[{\mathrm{Al}}_{2}O_{3}\cdot 2\mathrm{Si}O_{2}]\;(s)+0.5[{\gamma -\mathrm{Al}}_{2}O_{3}]\;(s)$$

Due to the unavailability of data on metakaolinite, the phase transformation temperature of kaolinite during thermal activation is inferred from experimental findings reported by literature [59,60]. The transition temperature from kaolinite to metakaolinite ranges between 500 °C and 700 °C [Equation (9)].

**Figure 7:**
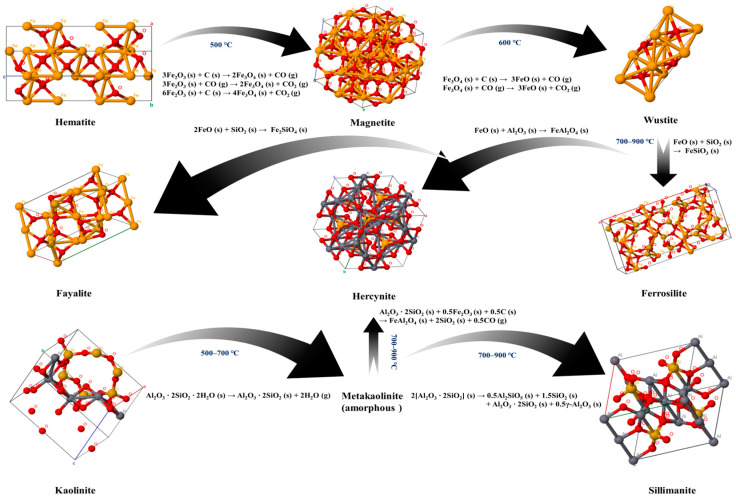
Mechanism of co-roasting of CG and CGCS.

The diagram in Figure 7 illustrates the mechanism of co-roasting CG and CGCS. During the co-roasting process, hematite (Fe_2_O_3_) is reduced to magnetite (Fe_3_O_4_), which further undergoes reduction to wustite (FeO) due to the presence of fixed carbon in CG and CGCS. Subsequently, wustite (FeO) reacts with Si and Al elements in the mixed raw material, resulting in the formation of iron phases such as ferrosilite (FeSiO_3_), hercynite (FeAl_2_O_4_), and fayalite (Fe_2_SiO_4_). Additionally, kaolinite present in the mixed raw material undergoes dehydration into metakaolinite, which then transforms into sillimanite (Al_2_SiO_5_) and hercynite (FeAl_2_O_4_). In summary, CGCS enables efficient reduction of iron minerals in CG while also enhancing leaching rates of Al^3+^ and TFe.

### 2.5. Optimization of H_2_SO_4_ Leaching Parameter

After conducting the co-roasting experiment, a significant enhancement in the activity of aluminum and iron was observed. Consequently, batch experiments were conducted to investigate the optimal leaching conditions, encompassing H_2_SO_4_ concentration and leaching temperature.

**Figure 8:**
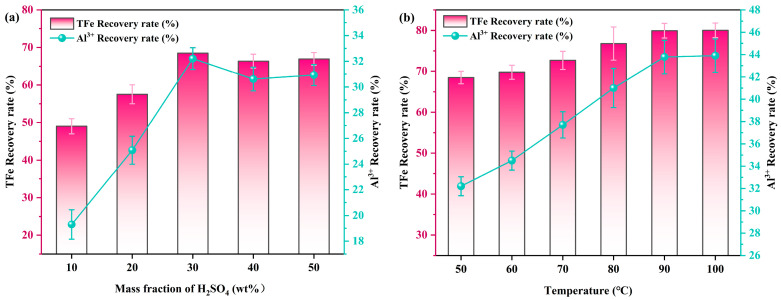
(**a**) The effect of H_2_SO_4_ concentration on the leaching rates of Al^3+^ and TFe, (**b**) the effect of leaching temperature on the leaching rates of Al^3+^ and TFe (co-roasting process: CG/CGCS mass ratio of 8/2, 600 °C, 1 h; H_2_SO_4_ leaching process: 5 h, liquid to solid ratio of 5:1 mL/g).

As can be seen from Figure 8a, the effect of H_2_SO_4_ concentration (10–50 wt%) on the extraction of valuable metal elements were investigated under this conditions, including a CGCS content of 20 wt%, co-roasting in a tubular pyrolysis furnace at 600 °C for 1 h, leaching temperature of 50 °C, liquid to solid ratio of 5:1 mL/g, and leaching time of 5 h. With the increase in H_2_SO_4_ concentration, the leaching rates of Al^3+^ and TFe exhibited an initial upward trend followed by a plateau. Specifically, at an H_2_SO_4_ concentration of 30 wt%, the leaching rate of TFe was measured to be 68.47%, while that of Al^3+^ was found to be 32.21%. Subsequently, further increases in H_2_SO_4_ concentration only resulted in marginal changes in the leaching rates of Al^3+^ and TFe, indicating near-complete reaction between Al^3+^, TFe, and H_2_SO_4_ at a concentration of 30 wt%. Therefore, it can be inferred that co-roasting pretreatment with CG and CGCS achieves optimal leaching efficiency when using an H_2_SO_4_ concentration of 30 wt%.

The effect of leaching temperature (50 °C–100 °C) on the extraction of valuable metal elements were investigated, as depicted in Figure 8b. In this study, the H_2_SO_4_ concentration was fixed at 30 wt%, while other conditions such as the liquid to solid ratio were maintained constant. As shown in Figure 8b, the leaching rates of Al^3+^ and TFe at 50 °C were only 32.21% and 68.47%, respectively. As the temperature increased, both the leaching rates of Al^3+^ and TFe gradually improved; when the temperature reached 90 °C, the leaching rate of TFe reached 79.93%, while that of Al^3+^ was recorded as 43.78%. This can be attributed to enhanced diffusion processes and chemical reaction rates with increasing temperature, leading to more complete reactions. Moreover, beyond a temperature of 90 °C, further increases had minimal impact on enhancing the leaching rates of Al^3+^ and TFe significantly. Therefore, a leaching temperature of 90 °C was selected.

### 2.6. Analysis of Mixed Raw Material, Co-Roasting Product and Leached Residue

**Figure 9:**
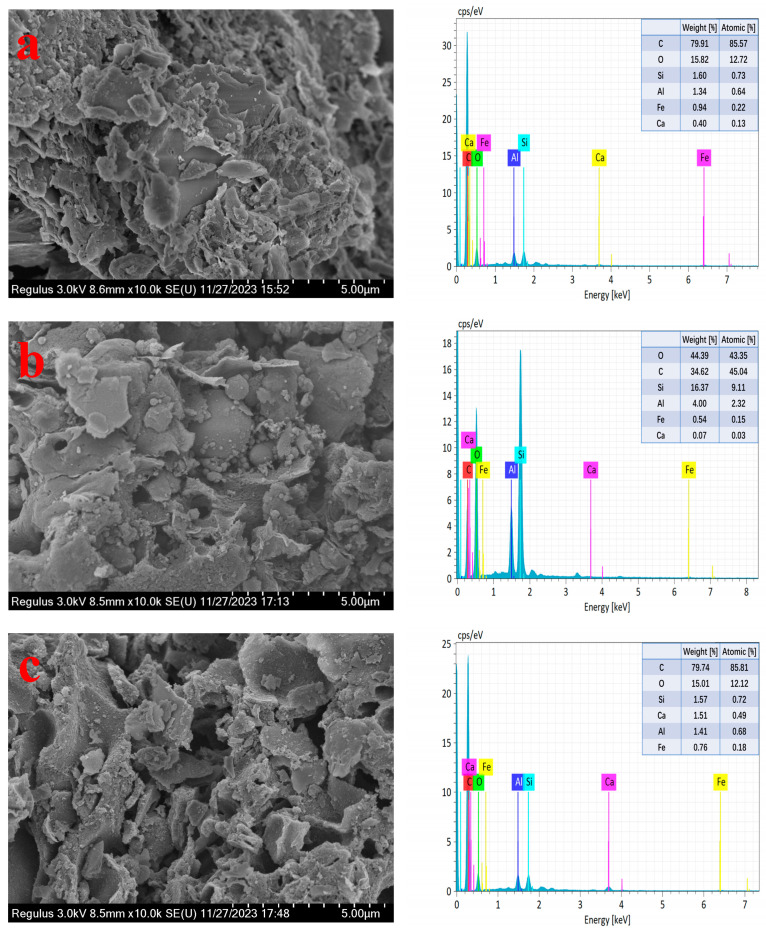
SEM and EDS images of mixed raw material (**a**), co-roasting product (**b**), and leached residue (**c**).

The mixed raw material, as well as the co-roasting product and leached residue obtained under optimal conditions, were characterized to analyze their differences. The SEM-EDS images of the three samples are recorded and presented in Figure 9. As depicted in Figure 9a, the particle size distribution of the mixed raw material exhibits non-uniformity, while displaying a typical lamellar structure of kaolinite. In Figure 9b, it can be observed that despite dehydroxylation of kaolinite into metakaolinite during co-roasting, the lamellar structure still exists, which may be attributed to the fact that although the AlO_2_(OH)_4_ octahedral layer is damaged, the SiO_4_ tetrahedral layer can still maintain the lamellar structure. Furthermore, Figure 9c presents a scanning electron microscope image of the leached residue revealing evident signs of erosion. This indicates that after leaching aluminum and iron with sulfuric acid, the microstructure of aluminosilicates is disrupted. Additionally, both co-roasting product and leached residue exhibit some pore structures.

The EDS diagram in Figure 9 reveals a significantly higher mass percentage of elemental Ca in the leached residue compared to the other two samples, which may be due to the reaction of sulphuric acid with CaO in the samples to generate calcium sulphate, resulting in the enrichment of calcium ions in the leached residue.

**Figure 10:**
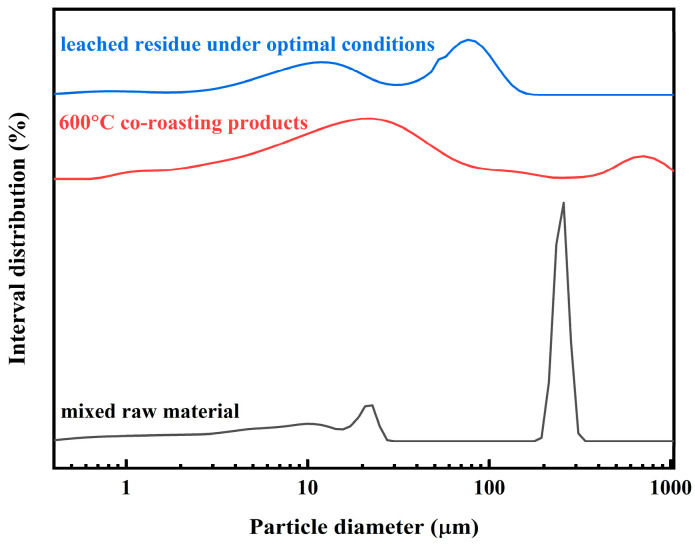
The particle size distribution of the mixed raw material, co-roasting product, and leached residue.

The particle size distribution of the mixed raw material, co-roasting product, and leached residue is illustrated in Figure 10. The average particle sizes of the mixed raw material, co-roasting product, and leached residue were determined to be 151.10 μm, 103.87 μm, and 42.86 μm, respectively. Notably, the leached residue exhibited the smallest average particle size among all samples investigated, consistent with SEM observations and further confirming its abundant pore structure. 

### 2.7. Prospects and Challenges in the Application of Acid Solution and Leached Residue

Table 1 illustrates the Chemical composition (wt%) of leached residue and chemical content (g/L) of an acid solution. As given in Table 1, alumina, potassium oxide, iron oxide and sodium oxide from the co-roasting product were partially dissolved in the acid solution, while calcium oxide and magnesium oxide reacted with sulphuric acid and were deposited in the leached residue, and the leached residue had the largest percentage of SiO_2_; this is in agreement with the results of XRD analyses (Figure S3) of the leached residue.

Most of the existing coal-based waste treatment technologies focus solely on extracting valuable metal elements from a single type of coal-based waste residue, neglecting the synergistic extraction potential of multiple types of coal-based waste residues. In this study, a combination of CG and CGCS co-roasting + H_2_SO_4_ leaching was employed to extract valuable components (Al, Fe) from the acid solution, resulting in a leached residue with high silicon content and abundant pore structures. This indicates that both the acid solution and leached residue obtained in this study possess rich usable resources; however, effectively harnessing their value while minimizing environmental pollution remains a significant challenge. The technologies for disposal of coal-based waste residues have been systematically summarized and compared, with results presented in Table 2. 

We believe that the findings of these studies highlight the potential applications and benefits of utilizing H_2_SO_4_ acid solution and coal-based waste leached residue, providing technical support for the proper handling of acid solution and leached residue. It is crucial to note that adherence to regulations and guidelines is essential in handling these leaching wastes to minimize their environmental impact. Furthermore, our next step involves utilizing aluminum and iron ions present in the acid solution for fabricating coal-based waste residues-based aluminum-iron flocculants, which can be applied in treating industrial wastewater such as coal slurry water and dye wastewater, with a view to achieving the purpose of treating wastes with wastes. Simultaneously, we also focus on preparing mesoporous materials from acid-leached residue, with subsequent systematic investigations planned.

## 3. Materials and Methods

### 3.1. Materials

The CG and CGCS used in this study were obtained from Yan’an City, Shaanxi Province, China. The CG and CGCS were crushed and then ground with a rod mill for 2–3 min, passing through a 100-mesh sieve. The used H_2_SO_4_ was of analytical reagent and was purchased from Xi ‘an Jiuheng Huaye Equipment Co., Ltd. The experiments were conducted using deionized water.

The chemical compositions of CG and CGCS, as well as their proximate analysis, are presented in Table 3.

### 3.2. Experimental Procedures

**Figure 11:**
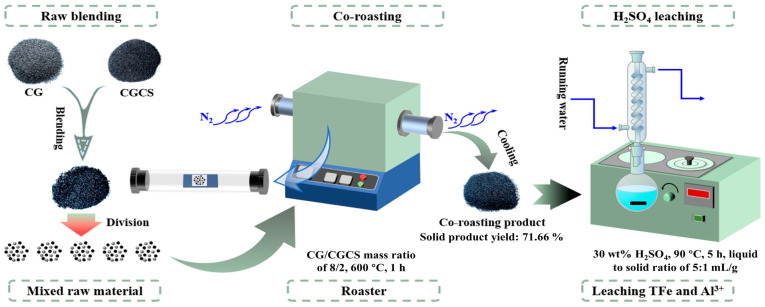
Schematic of CG and CGCS co-roasting experiment.

The schematic in Figure 11 illustrates the co-roasting experiment of CG and CGCS, consisting of two main steps: co-roasting and H_2_SO_4_ leaching. In a typical co-roasting experiment, the as-received CG was thoroughly mixed with CGCS in a specific ratio, placed flat in a corundum crucible, and subjected to co-roasting at predetermined temperatures (500 °C–900 °C) and duration (1 h) using a tubular pyrolysis furnace (model GSL-1500X). The tubular pyrolysis furnace is filled with N_2_ gas to provide a reduced atmosphere. To investigate the effect of co-roasting+H_2_SO_4_ leaching conditions on the extraction efficiency of valuable metal elements, the leaching process was conducted under the following conditions: leaching temperatures ranging from 50 °C to 100 °C, H_2_SO_4_ concentration varying from 10 wt% to 50 wt%, liquid to solid ratio set at 5:1 mL/g, and a leaching time of 5 h. The optimization process of the above experimental conditions is shown in Figures S1 and S2. The co-roasting product was subjected to H_2_SO_4_ leaching in a flat-bottomed flask equipped with a condensing device.

Firstly, the co-roasting product obtained under optimal co-roasting conditions was mixed with predetermined amounts of H_2_SO_4_ at a specified liquid to solid ratio. Subsequently, the resulting slurry was transferred to a flat-bottomed flask equipped with a condensing device and subjected to reaction in a thermostatic magnetic water bath pot, while maintaining a stirring speed of 150 r/min. Once the temperature of the mixed slurry reaches the predetermined temperature, the timing for the reaction begins. After the reaction, the slurry is cooled to room temperature and filtered. Evaluate the concentration of Al^3+^ and total iron (TFe) in the leaching solution, determine the ratio of Al^3+^ and TFe in the leaching solution to that in the mixed raw material, and subsequently calculate the leaching efficiency. These indicators of aluminum and iron leaching rates in the leaching solution serve as performance assessment metrics for evaluating the effectiveness of the co-roasting process.

The equation for determining the leaching rate is as follows:

(12) $$\eta_{{\mathrm{Al}}^{3+}/\mathrm{TFe}}=\frac{m}{M}\;\times \;100\%$$

where η represents the recovery rates of Al^3+^ and TFe, while m and M denote the Al^3+^ and TFe contents in the leaching solution and mixed raw material, respectively.

### 3.3. Characterization Methods

The chemical compositions of both the CG, CGCS, and leached residue were analyzed by X-ray fluorescence (XRF, Tokyo Rigaku Co., Simultix 12, Tokyo, Japan). XRF analysis was conducted with an Rh target, Si (Li) crystal detector, and calibrated based on fundamental parameters. Proximate analysis of the CG and CGCS was performed according to GB/T 212-2008 standards [67]. The pyrolysis characteristics of CG and mixed raw material were recorded using a thermogravimetric analyzer (TGA 8000 Perkin Elmer, Waltham, MA, USA) from 35 °C to 1000 °C in a nitrogen atmosphere at a flow rate of 20 mL/min. The heating rates of 10 °C/min were set to research the pyrolysis characteristics. The mineral phase-evolution features of the samples were conducted by X-ray diffraction technique (XRD; Bruker D8 ADVANCE, Ettlingen, Germany) with Cu Kα radiation from 10° to 80° at a scan speed of 10 °/min. The chemical bonds and functional groups in the samples were analyzed through a Fourier transform infrared spectrometer (FTIR; Bruker VERTEX 70, Ettlingen, Germany) within the wavenumber range of 400–3800 cm^−1^. XPS analysis was performed on a ULVAC Inc. spectrometer equipped (XPS, ULVAC PHI 5000 VersaProbe 4, Chigasaki, Japan) with monochromatic Al Kα radiation as an excitation source, and the analyzed area of 400 μm in diameter. Raw data obtained were processed by the XPS Peak Fits software package (version number: XPSpeak 41) to fit all spectra. A scanning electron microscope (JSM-6390A, Jeol, Tokiyo, Japan) was used to analyze the microscopic morphology and the type and percentage of elements of samples. The particle size distribution of the samples was determined using a laser particle size analyzer (EyeTech, S/N 60296, Carlton, VIC, Australia). Analysis of the composition of the leaching solution was performed by using spectrophotometry. 

## 4. Conclusions

The present study proposes an innovative approach of co-roasting and H_2_SO_4_ leaching to extract valuable metal elements (Al, Fe) from CG and CGCS, aiming at their treatment. Through thermodynamic analyses and systematic experimental investigations, the following outcomes were obtained:

(1) The effects of various factors on the extraction efficiency of valuable metal elements (Al, Fe) were investigated through single factor experiments. Under optimal conditions including 20 wt% CGCS content, 600 °C co-roasting temperature for 1 h followed by leaching at 90 °C with a liquid to solid ratio of 5:1 mL/g using a H_2_SO_4_ concentration of 30 wt%, a TFe leaching rate of 79.93% was achieved along with an Al^3+^ leaching rate of 43.78% after a leaching time of 5 h. Furthermore, we found that acid solution and leached residue both have broad application prospects.
(2) The activation behavior and phase transformation mechanism during the co-roasting process were investigated through Gibbs free energy calculation, as well as XRD, FTIR, and XPS characterization analysis. Inert kaolinite in CG and CGCS was converted to active metakaolinite after co-roasting. The reaction of metakaolinite with Si and Al elements from the mixed raw material produced sillimanite (Al_2_SiO_5_) and hercynite (FeAl_2_O_4_). Hematite (Fe_2_O_3_) was reduced to magnetite (Fe_3_O_4_) and wustite (FeO) by fixed carbon in CG and CGCS. Subsequently, wustite reacted with Si and Al elements from the mixed raw material to form iron phases such as ferrosilite (FeSiO_3_), hercynite (FeAl_2_O_4_), and fayalite (Fe_2_SiO_4_).
(3) Co-roasting+H_2_SO_4_ leaching provides a novel method for extensive utilization of CG and CGCS while also offering a new approach for treating two or more types of coal-based solid waste that can alleviate industry development’s pressure on the environment.

## Figures and Tables

**Table 1:** Chemical composition (wt%) of leached residue and chemical content (g/L) of an acid solution.

	SiO_2_	Al_2_O_3_	K_2_O	Fe (Fe_2_O_3_, Fe_3_O_4_, FeO, etc.)	S (CaSO_4_, MgSO_4_, etc.)	Na_2_O	Others
The chemical composition (wt%) of leached residue	74.29	13.44	3.31	1.34	0.524	0.492	6.604
	Al_2_(SO_4_)_3_		FeSO_4_	Fe_2_(SO_4_)_3_	K_2_SO_4_	Na_2_SO_4_	Others
The chemical content (g/L) of an acid solution	0.82		0.92	0.09	0.02	0.01	<0.01

**Table 2:** Summary and comparison of coal-based waste residues disposal technologies.

Substance	Methods	Reagents	Extraction	Application	Ref.
Acid solution (high-iron CG)	Calcination + acid leaching	HCl	Al: 90% Fe: 91%	Preparation of PAFC flocculants	[61]
Acid solution (high-alumina fly ash)	Acid leaching + direct-electricity conversion technology + roasting	HCl	No reported	Preparation of Al_2_O_3_	[62]
Aacid solution (coal-bearing kaolinite)	Mechanical grinding + acid leaching	H_2_SO_4_	Al: 100%	Preparation of γ-Al_2_O_3_ powder	[63]
Leached residue (CG)	Calcination + acid leaching	HCl	No reported	Preparation of NaA zeolite	[64]
Leached residue (CGFS)	Calcination + acid leaching	HCl	The total leaching rate of all metal oxides: 80%	Preparation of mesoporous silica	[65]
Leached residue (CGS)	Non-hydrothermal sol–gel method	No reported	No reported	Preparation of MCM-41	[66]
Acid solution and leached residue (CG)	High temperature acid leaching	HCl	Al: 92.54% SiO_2_: 96.01%	Preparation of Al_2_O_3_ and SiC	[15]
Acid solution and leached residue (CG and CGCS)	Co-roasting + acid leaching	H_2_SO_4_	Al: 43.78% Fe: 79.93%	Preparation of aluminium-iron flocculants and mesoporous silica	This work

**Table 3:** Chemical composition and proximate analysis of CG and CGCS (mass, %).

	Substance	SiO_2_	Al_2_O_3_	Fe_2_O_3_	K_2_O	MgO	CaO	Others	LOI
Chemical composition	Content in CG	58.02	20.87	10.67	4.41	2.17	1.34	2.52	20.10
	Content in CGCS	28.80	10.51	20.23	1.37	2.36	31.93	4.80	36.75
	Substance	Moisture		Ash		Volatiles		Fixed carbon	
Proximate analysis	Content in CG	1.7		85.81		9.93		2.56	
	Content in CGCS	2.15		74.88		5.78		17.19	

The Sulphur content of CG and CGCS both are <0.1%.

## Data Availability

Data are contained within the article and Supplementary Materials.

## Supplementary Materials

The following supporting information can be downloaded at: https://www.mdpi.com/article/10.3390/molecules29010130/s1, Figure S1: Coal gangue H_2_SO_4_ leaching experiments; Figure S2: Coal gangue calcination+H_2_SO_4_ leaching experiments; Figure S3: XRD patterns of leached residue; 1-kaolinite; 2-quartz; 3-hematite; 4-muscovite; 5-calcite; 6-magnetite; 7-wustite; 8-fayalite; 9-sillimanite; 10-hercynite; 11-ferrosilite; 12-magnesium sulfate; 13-calcium sulfate; 14-potassium oxide; 15-alumina oxide; 16-sodium oxide.
