# Supplementary material for: Study on Extraction Valuable Metal Elements by Co-Roasting Coal Gangue with Coal Gasification Coarse Slag
Source: Molecules. 2023 Dec 25;29(1):130. doi: 10.3390/molecules29010130 (PMC10779775; doi:10.3390/molecules29010130)
Supplement: Supplementary file 1 [file molecules-29-00130-s001.zip › molecules-2670740-supplementary.pdf]

Supplementary Information

Study on Extraction Valuable Metal Elements by Co-Roasting Coal Gangue with Coal Gasification Coarse Slag

Jincheng Zhao [1,2,3], Tao Yu [1,2,3,*], Huan Zhang [1], Yu Zhang [1], Lanting Ma [1,3], Jinling Li [2,3], Chengtun Qu [1,2] and Te Wang [2,3]

[1] Yan'an Key Laboratory of Low Carbon Synergistic Control Technology and Reservoir Protection for Oil and Gas Field Environmental Pollution, Shaanxi Fuquan Environmental Protection Technology Co., Ltd., Yan'an 727500, China; 21211070886@stumail.xsyu.edu.cn (J.Z.); zhuan@wnu.edu.cn (H.Z.); zhangyu@xynu.edu.cn (Y.Z.); malanting2020@xsyu.edu.cn (L.M.); xianqucth@xsyu.edu.cn (C.Q.)
[2] State Key Laboratory of Petroleum Pollution Control, College of Chemistry and Chemical Engineering, Xi'an Shiyou University, Xi'an 710065, China; lijinling@xsyu.edu.cn (J.L.); 21212070982@stumail.xsyu.edu.cn (T.W.)
[3] Shaanxi Oil and Gas Pollution Control and Reservoir Protection Key Laboratory, College of Chemistry and Chemical Engineering, Xi'an Shiyou University, Xi'an 710065, China
* Correspondence: 180708@xsyu.edu.cn

1. Experimental procedures

To investigate the effect of co-roasting+H_2SO_4 leaching conditions on the extraction efficiency of valuable metal elements, the optimization of experimental conditions of coal gangue H_2SO_4 leaching experiments and coal gangue calcination+H_2SO_4 leaching experiments were carried out in turn, and the optimization process is as follows.

1.1. Coal gangue H_2SO_4 leaching experiments

Firstly, the coal gangue was mixed with predetermined amounts of H_2SO_4 at a specified liquid to solid ratio. Subsequently, the resulting slurry was transferred to a flat-bottomed flask equipped with a condensing device and subjected to reaction in a thermostatic magnetic water bath pot, while maintaining a stirring speed of 150 r/min. Once the temperature of the mixed slurry reaches the predetermined temperature, the timing for the reaction begins. After the reaction, the slurry is cooled to room temperature and filtered. Evaluate the concentration of Al^{3+} and total iron (TFe) in the leaching solution, determine the ratio of Al^{3+} and TFe in the leaching solution to that in the raw material, and subsequently calculate the leaching efficiency. These indicators of aluminum and iron leaching rates in the leaching solution serve as performance assessment metrics for evaluating the effectiveness of the H_2SO_4 leaching process.

The equation for determining the leaching rate is as follows:

$$\eta_{Al^{3+}/TFe} = \frac{m}{M} \times 100\% \quad (1)$$

where η represents the recovery rates of Al^{3+} and TFe, while m and M denote the Al^{3+} and TFe contents in the leaching solution and raw material, respectively.

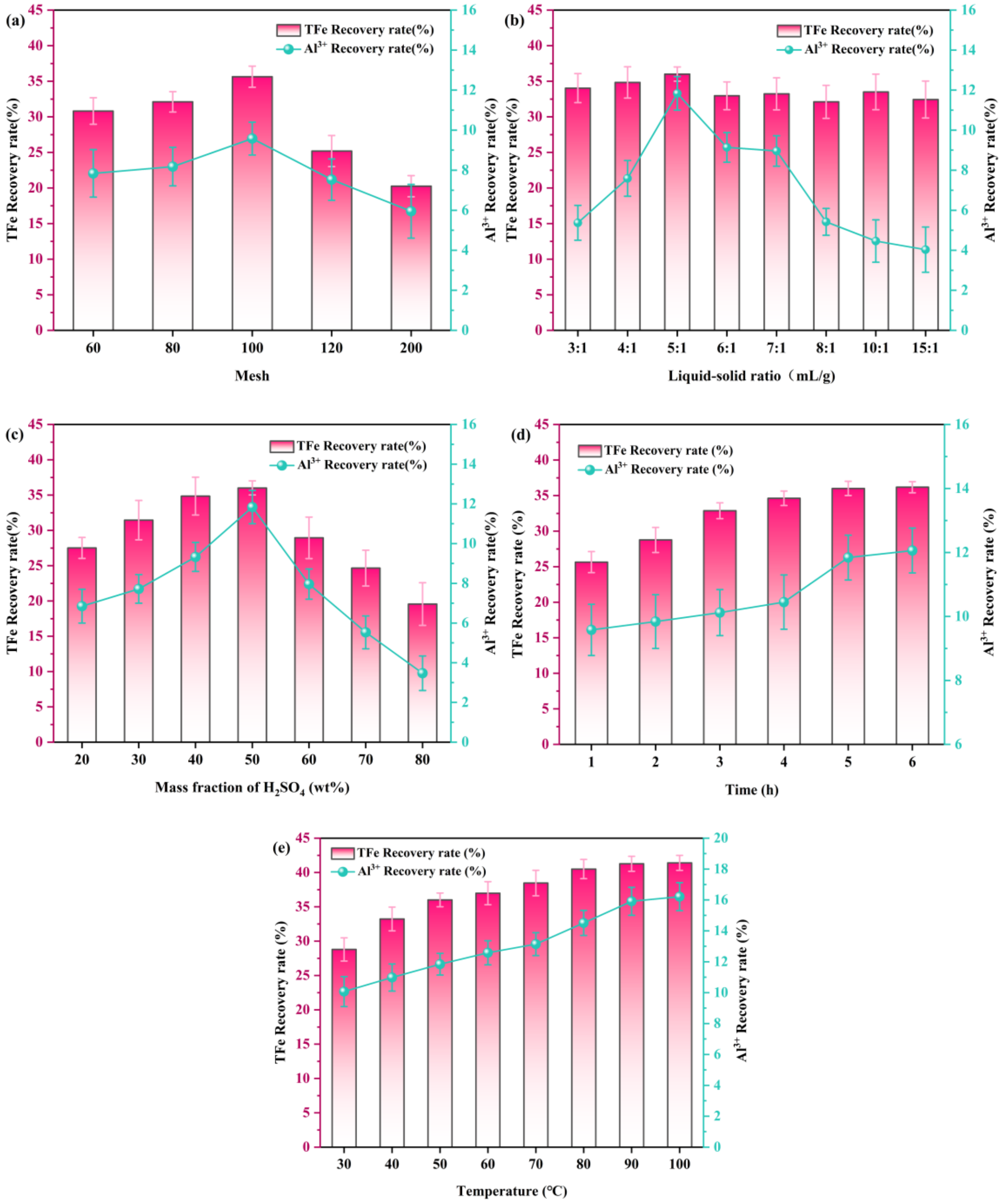

Figure S1. Coal gangue H_2SO_4 leaching experiments.

As depicted in Figure S1, the optimum experimental conditions for H_2SO_4 leaching experiments of coal gangue were 100 mesh, liquid to solid ratio of 5:1 mL/g, H_2SO_4 concentration of 50 wt%, a leaching time of 5 h, and a leaching temperature of 90 °C.

1.2. Coal gangue calcination+H_2SO_4 leaching experiments

In order to investigate the effect of coal gangue calcination+H_2SO_4 leaching conditions on the extraction efficiency of valuable metal elements, the following four experimental conditions were optimized, in which the particle size, liquid-solid ratio and H_2SO_4 leaching time were taken from the results of acid leaching experiments of coal gangue (the coal gangue calcination experiment involves subjecting a certain quantity of coal gangue to different temperatures in an air atmosphere within a muffle furnace for a designated duration).

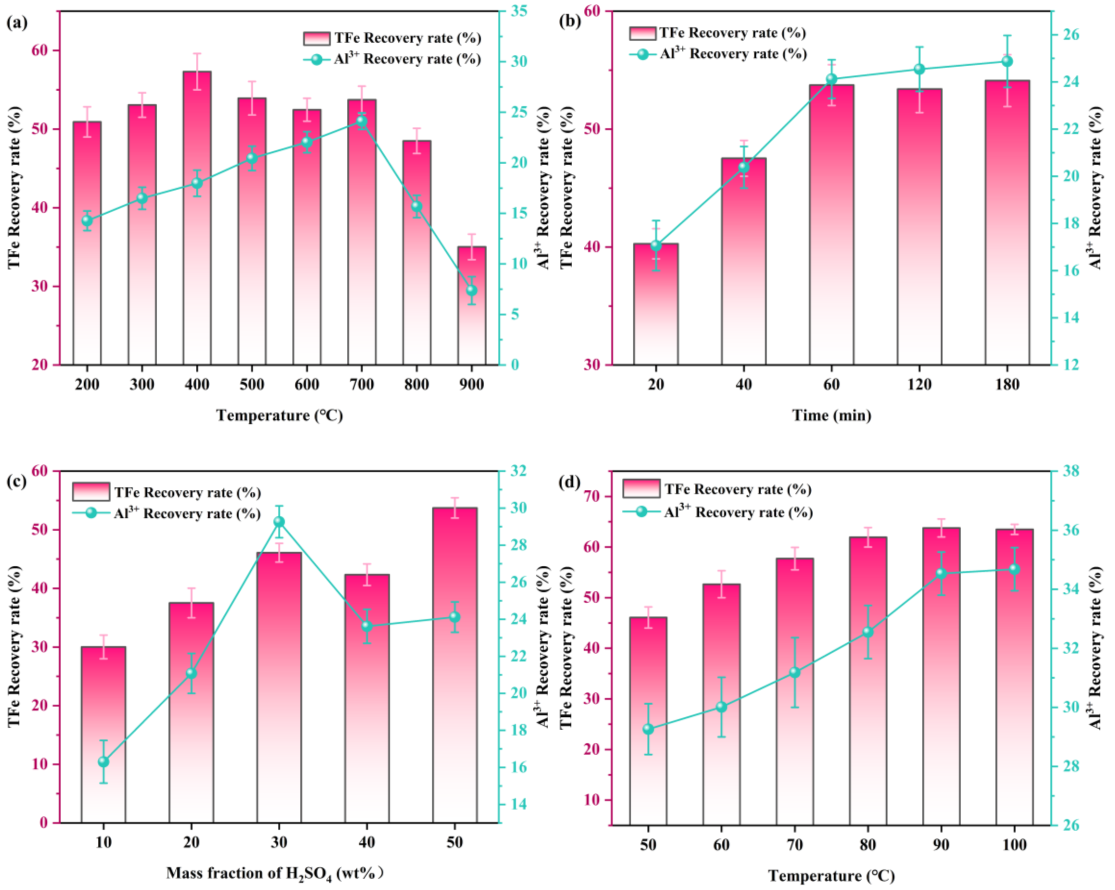

Figure S2. Coal gangue calcination+H_2SO_4 leaching experiments.

Figure S2 shows the optimum experimental conditions for calcination+H_2SO_4 leaching experiments of coal gangue were 100 mesh, liquid to solid ratio of 5:1 mL/g, a leaching time of 5 h, calcination temperature of 700 °C, calcination time of 1 h, H_2SO_4 concentration of 30 wt%, and a leaching temperature of 90 °C.

In summary, the optimization of the aforementioned experimental conditions ultimately led to the identification of optimal parameters for conducting co-roasting+H_2SO_4 leaching experiments (100 mesh, liquid to solid ratio of 5:1 mL/g, a leaching time of 5 h, and a co-roasting time of 1 h).

2. XRD patterns of leached residue

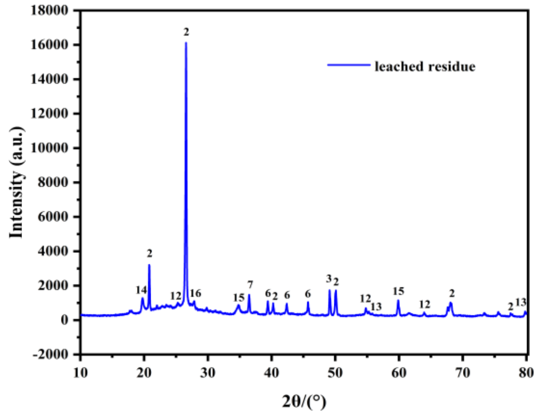

Figure S3. XRD patterns of leached residue; 1-kaolinite; 2-quartz; 3-hematite; 4-muscovite; 5-calcite; 6-magnetite; 7-wustite; 8-fayalite; 9-sillimanite; 10-hercynite; 11-ferrosilite; 12-magnesium sulfate; 13-calcium sulfate; 14-potassium oxide; 15-alumina oxide; 16-sodium oxide.

The XRD analysis revealed diffraction peaks corresponding to quartz, hematite, magnetite, wustite, magnesium sulfate, calcium sulfate, potassium oxide, alumina oxide, sodium oxide, and other compounds, which are consistent with the chemical composition of the leached residue.
